# Report tau or exp(tau) rather than tau-squared in random-effects meta-analyses

**DOI:** 10.1017/rsm.2026.10075

**Published:** 2026-02-25

**Authors:** Mark D. Chatfield, Louise Marquart-Wilson, Annette Dobson, Daniel Farewell

**Affiliations:** 1 School of Public Health, The University of Queensland, Australia; 2 School of Medicine, Cardiff University, United Kingdom

**Keywords:** geometric mean, geometric standard deviation, heterogeneity, lognormal

## Abstract

In random-effects meta-analysis, the between-study heterogeneity variance, $\tau^{2}$ , is often reported but is not easy to interpret. For meta-analyses of differences (such as mean differences, standardized mean differences, or risk differences), the standard deviation (SD), τ , indicates the extent to which studies’ true effects vary about their average. For meta-analyses of (natural) log-transformed measures of effect (such as log risk ratios [RRs]), we explain how the geometric SD, exp⁡(τ) , is helpful to understand how *untransformed* measures (such as RRs) vary multiplicatively about their average. We recommend that authors and software developers report τ for differences and exp⁡(τ) for ratios, rather than $\tau^{2}$ . This will facilitate the interpretation of the *magnitude* of heterogeneity values, for example, the interpretation of heterogeneity estimates and confidence intervals beyond simple binary statements about the presence or absence of heterogeneity.

## Highlights

### What is already known?

In random-effects meta-analysis, the between-study heterogeneity variance, $\tau^{2}$ , is often reported but is not easy to interpret. For meta-analyses of differences, the standard deviation (SD), τ , is helpful to understand the extent to which studies’ true differences vary about their average.

### What is new?

For meta-analyses of ratios (such as odds ratios, risk ratios, etc.), the geometric SD, exp⁡(τ) , is helpful to understand the extent to which studies’ true ratios vary multiplicatively about their average.

### Potential impact for *RSM* readers

We recommend that authors and software developers report τ for differences and exp⁡(τ) for ratios, rather than $\tau^{2}$ . This will facilitate the interpretation of the *magnitude* of heterogeneity values, for example, the interpretation of heterogeneity estimates and confidence intervals beyond simple binary statements about the presence or absence of heterogeneity.

## 1 Introduction

In random-effects meta-analysis, the distribution of underlying true effect sizes is modeled. The extent of heterogeneity (i.e., how studies’ true effects vary about their average) is very important. For example, an estimate of heterogeneity can substantially influence the calculation of i) a confidence interval (CI) of the average effect^1^ and ii) a prediction interval for the true effect of the next study.^2^

Although heterogeneity values appear in various graphs, tables, and text,^1^^–^ ^4^ they are often expressed in a way that does not facilitate understanding. For example, the heterogeneity variance ( $\tau^{2}$ ) is reported more frequently than the standard deviation (SD, τ ).^5^ For meta-analyses of differences (such as mean differences, standardized mean differences [SMDs], or risk differences), τ is on the same scale and is easier to interpret.^5^ For meta-analyses of ratios (such as odds ratios, risk ratios [RRs], hazard ratios, incidence rate ratios, and ratios of means or response ratios), τ (the SD of log-transformed ratios) is on the logarithmic scale^6^ and is less obviously interpretable.

In this article, we explain how τ is helpful to understand the heterogeneity of differences and how exp⁡(τ) is helpful to understand the heterogeneity of ratios. We also explain how some values of τ itself can be meaningfully interpreted for ratios in an Appendix of the Supplementary Material. We recommend that authors and software developers replace the reporting of $\tau^{2}$ with more accessible formulations of heterogeneity. This will facilitate the interpretation of the *magnitude* of heterogeneity values, for example, the interpretation of heterogeneity estimates and CIs beyond simple binary statements about the presence or absence of heterogeneity.

## 2 Random-effects models

### 2.1 Differences

We consider a meta-analysis model of difference measures of effect, with a focus on SMDs. Let $\theta_{i}$ denote the true SMD for study i (i=1,…,k) . A random-effects model for SMDs can be described in terms of the true SMD $\theta_{i}$ , the observed SMD ${\hat{\theta }}_{i}$ , and its standard error $\sigma_{i}$ . A popular model^6^ is

(1) $$\begin{array}{rl} {\hat{\theta }}_{i} & ∼N(\theta_{i},\sigma_{i}^{2}), \end{array}$$

(2) $$\begin{array}{rl} \theta_{i} & ∼N(\mu ,\tau^{2}). \end{array}$$

The modeled distribution of true SMDs is a normal distribution with mean μ and SD τ . Therefore, the interval [μ−τ,μ+τ] covers approximately 68% (or 2/3) of the distribution and the interval [μ−2τ,μ+2τ] covers approximately 95% (or 19/20) of the distribution (as does the interval [μ−1.96τ,μ+1.96τ] ). We will refer to such intervals as 68% and 95% ranges.

### 2.2 Ratios

We now consider a meta-analysis model of ratio measures of effect, with a focus on RRs. Throughout, log denotes the natural logarithm.

Let $\alpha_{i}$ denote the true RR for study i (i=1,…,k) . A random-effects model for RRs is typically described in terms of the true log RR ( $\theta_{i}=\log\alpha_{i}$ ), the observed log RR ( ${\hat{\theta }}_{i}$ ), and its standard error ( $\sigma_{i}$ ) using (1) and (2).

We now focus on describing the modeled distribution of true RRs, $\alpha_{i}=\exp(\theta_{i})$ . A lognormal distribution is implied, and the geometric mean (GM) and median of the distribution are exp⁡(μ) . A pooled or overall RR from a meta-analysis is an estimate of the GM . There are several ways to describe variation about the GM .^7^^,^ ^8^ We explain the simplest way by using exp⁡(τ) below, and an alternative way using τ directly in the Appendix of the Supplementary Material.

The geometric SD of the modeled distribution of true RRs is exp⁡(τ) . It quantifies variation about the GM in a multiplicative manner.^7^ Approximately 68% of the distribution lies in the interval [LB1,UB1] , where

$$\begin{array}{rl} \mathrm{LB}1 & =\exp(\mu -\tau )=\exp(\mu )\times \exp(-\tau )=\mathrm{GM}/\exp(\tau ),\\ \mathrm{UB}1 & =\exp(\mu +\tau )=\exp(\mu )\times \exp(\tau )=\mathrm{GM}\times \exp(\tau ). \end{array}$$

Approximately 95% of the distribution lies in the interval [LB2,UB2] , where

$$\begin{array}{rl} \mathrm{LB}2 & =\exp(\mu -2\tau )=\exp(\mu )\times \exp(-2\tau )=\mathrm{GM}/\{\exp(\tau ){\}}^{2},\\ \mathrm{UB}2 & =\exp(\mu +2\tau )=\exp(\mu )\times \exp(2\tau )=\mathrm{GM}\times \{\exp(\tau ){\}}^{2}. \end{array}$$

### 2.3 Prediction intervals

Reporting a prediction interval for the true effect of the next study^2^^,^ ^9^ is recommended by many.^5^^,^ ^10^^,^ ^11^ Most software packages will calculate and display a prediction interval on a forest plot.

Assuming that

(3) $$\begin{array}{rl} \theta_{k+1} & ∼N(\mu ,\tau^{2}), \end{array}$$

(4) $$\begin{array}{rl} \hat{\mu } & ∼N(\mu ,\mathrm{SE}(\hat{\mu }{)}^{2}), \end{array}$$

(5) $$\begin{array}{rl} \theta_{k+1}-\hat{\mu } & ∼N(0,\tau^{2}+\mathrm{SE}(\hat{\mu }{)}^{2}). \end{array}$$

Higgins et al.^2^ proposed an approximate 95% prediction interval for $\theta_{k+1}$ is

$$\begin{array}{r} \hat{\mu }\pm t_{k-2}\sqrt{\left\{{\hat{\tau }}^{2}+\hat{SE}(\hat{\mu }{)}^{2}\right\}}, \end{array}$$

where $t_{k-2}$ is the 0.975 quantile of the *t*-distribution with k−2 degrees of freedom. A 95% prediction interval calculated this way will be similar to the 95% range when $\hat{\mu }\approx \mu$ , $\hat{\tau }\approx \tau$ , the number of studies in a meta-analysis is not small, and $\hat{SE}(\hat{\mu }{)}^{2}<<{\hat{\tau }}^{2}$ .

However, a prediction interval will not convey the (often considerable) uncertainty in the estimate of τ . Therefore, a 95% CI for τ provides valuable information in addition to a prediction interval.^2^

## 3 Examples

### 3.1 Differences

**Figure 1:**
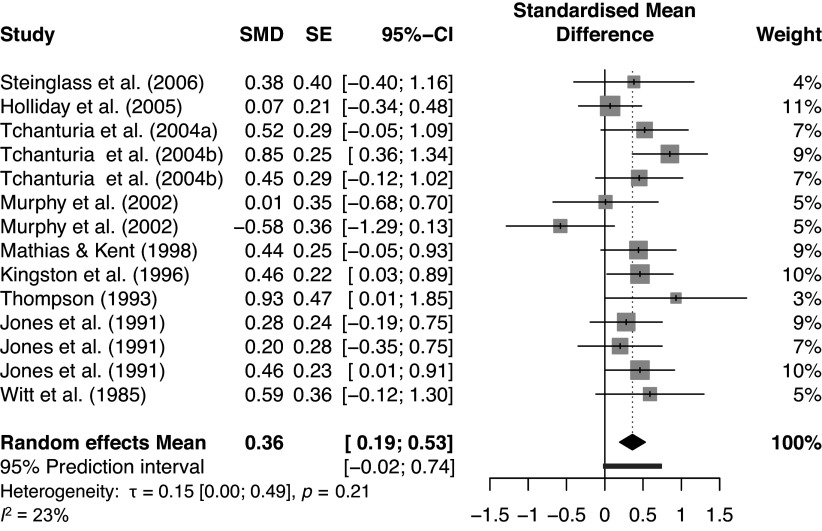
Random-effects meta-analysis comparing the time to complete a trail making task in people with eating disorders and healthy controls.^2^^,^ ^12^ DerSimonian and Laird estimator of τ used. Figure produced using the R package meta. Figure 1 Long description.

Roberts et al.^2^^,^ ^12^ performed meta-analysis on 14 studies comparing the time to complete a trail making task between people with eating disorders and healthy controls. They calculated SMDs (Cohen’s *d*) and considered these effect sizes negligible if ≥−0.15 and <0.15 , small if ≥0.15 and <0.40 , medium if ≥0.40 and <0.75 , large if ≥0.75 and <1.10 , very large if ≥1.10 and <1.45, and huge if ≥1.45 . We performed a random-effects meta-analysis on the data and produced a forest plot showing an estimate and 95% CI for τ (Figure 1).

In this example, the mean [95% CI] of the modeled distribution of true SMDs was estimated to be $\hat{\mu }=0.36\;[0.19,0.53]$ . The estimated SD of that distribution was $\hat{\tau }=0.15$ , which corresponds to a 68% range of [μ−0.15,μ+0.15] and a 95% range of [μ−0.30,μ+0.30] when a normal distribution is assumed. For example, if μ was 0.35, then the 68% range would be [0.2,0.5] and the 95% range would be [0.05,0.65] . We view this as a substantial amount of heterogeneity in this context. The 95% CI^1^ for τ was [0, 0.49], indicating that a degenerate distribution (homogeneity) is possible, as is a distribution with a huge SD (if τ=0.49, then the 95% range is [μ−0.98,μ+0.98] ). It is clear that there is considerable uncertainty in the SD of this distribution. These interpretations are readily apparent because an estimate and CI for τ were reported. This provides a more informative and nuanced understanding than a binary statement, such as “heterogeneity was present ( $\hat{\tau }>0$ )” or “no evidence of heterogeneity was found ( p=0.21 ).”

### 3.2 Ratios

**Figure 2:**
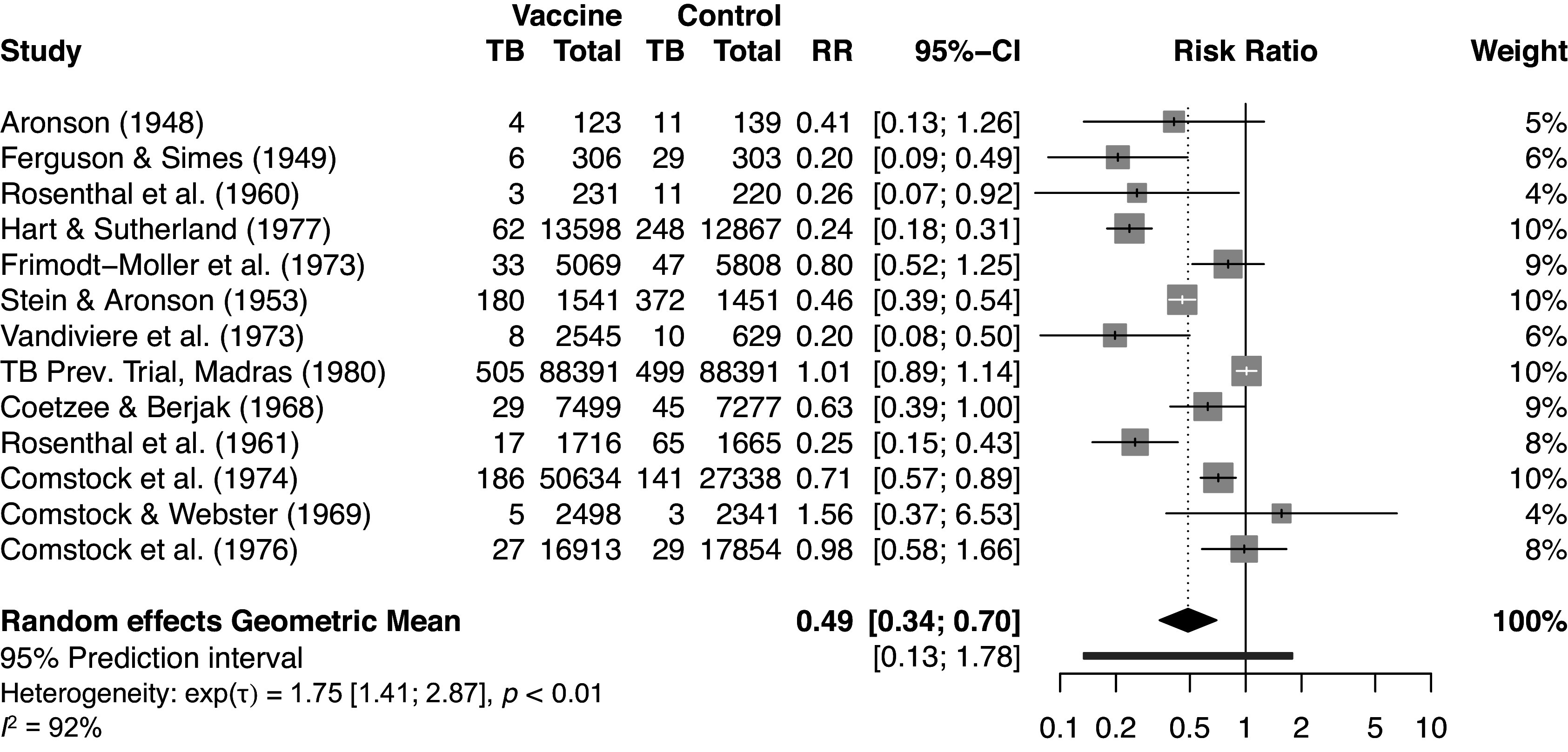
Random-effects meta-analysis comparing the risk of tuberculosis (TB) between vaccine and control groups.^13^ REML estimator of τ used. Figure produced using the R package meta with some manual editing. Figure 2 Long description.

The bacille Calmette–Guérin (BCG) vaccine is used to prevent tuberculosis. Colditz et al.^13^ performed a meta-analysis on the efficacy of the vaccine using RRs from 13 randomized trials. We performed a random-effects meta-analysis on the data and produced a forest plot showing an estimate and 95% CI for exp⁡(τ) (Figure 2).

The GM [95% CI] of the modeled distribution of true RRs was estimated to be $\hat{\mathrm{GM}}=0.49\;[0.34,0.70]$ . The estimated geometric SD was $\exp(\hat{\tau })=1.75$ , which corresponds to a 68% range of [GM/1.75,GM×1.75] and a 95% range of $\left[\mathrm{GM}/1.75^{2},\mathrm{GM}\times 1.75^{2}]=[\mathrm{GM}/3.06,\mathrm{GM}\times 3.06\right]$ when a lognormal distribution is assumed. For example, if the GM was 0.5, then the 68% range would be [0.29,0.88] and the 95% range would be [0.16,1.53] . We interpret this as considerable heterogeneity in the true RRs between trials. Repeating the process with the lower bound of the 95% CI^1^ for exp⁡(τ) (i.e., 1.41), if the GM was 0.5, then the 68% range would be [0.35,0.71] and the 95% range would be [0.25,0.99] . These calculations are straightforward because an estimate and CI of exp⁡(τ) were reported. Clearly, there is *much* heterogeneity here. This is more informative than a binary statement, such as “heterogeneity was present ( $\hat{\tau }>0$ )” or “evidence of heterogeneity was found ( p<0.01 ).”

## 4 Conclusion

For meta-analyses of differences, we recommend reporting τ rather than $\tau^{2}$ . For meta-analyses of ratios, we recommend reporting exp⁡(τ) rather than $\tau^{2}$ . This will facilitate the interpretation of the *magnitude* of heterogeneity estimates. Similarly, reporting CIs or credible intervals for τ or exp⁡(τ) will be more helpful than following the current recommendation to report intervals for $\tau^{2}$ .^1^^–^ ^3^

## Supporting information

10.1017/rsm.2026.10075.sm001 Chatfield et al. supplementary material Chatfield et al. supplementary material

## Data Availability

Previously published summary data are provided in the figures and in an Excel file.

## Supplementary material

The supplementary material for this article can be found at https://doi.org/10.1017/rsm.2026.10075.

## Long descriptions

### Figure 1 Long description

The forest plot contains columns for Study, S M D (Standardised Mean Difference), S E (Standard Error), 95 percent C I (Confidence Interval), a visual plot of the S M D, and Weight.

Individual study data from top to bottom:

* Steinglass et al. 2006: S M D 0.38, S E 0.40, C I -0.40 to 1.16, Weight 4 percent.

* Holliday et al. 2005: S M D 0.07, S E 0.21, C I -0.34 to 0.48, Weight 11 percent.

* Tchanturia et al. 2004a: S M D 0.52, S E 0.29, C I -0.05 to 1.09, Weight 7 percent.

* Tchanturia et al. 2004b: S M D 0.85, S E 0.25, C I 0.36 to 1.34, Weight 9 percent.

* Tchanturia et al. 2004b (second entry): S M D 0.45, S E 0.29, C I -0.12 to 1.02, Weight 7 percent.

* Murphy et al. 2002: S M D 0.01, S E 0.35, C I -0.68 to 0.70, Weight 5 percent.

* Murphy et al. 2002 (second entry): S M D -0.58, S E 0.36, C I -1.29 to 0.13, Weight 5 percent.

* Mathias and Kent 1998: S M D 0.44, S E 0.25, C I -0.05 to 0.93, Weight 9 percent.

* Kingston et al. 1996: S M D 0.46, S E 0.22, C I 0.03 to 0.89, Weight 10 percent.

* Thompson 1993: S M D 0.93, S E 0.47, C I 0.01 to 1.85, Weight 3 percent.

* Jones et al. 1991: S M D 0.28, S E 0.24, C I -0.19 to 0.75, Weight 9 percent.

* Jones et al. 1991 (second entry): S M D 0.20, S E 0.28, C I -0.35 to 0.75, Weight 7 percent.

* Jones et al. 1991 (third entry): S M D 0.46, S E 0.23, C I 0.01 to 0.91, Weight 10 percent.

* Witt et al. 1985: S M D 0.59, S E 0.36, C I -0.12 to 1.30, Weight 5 percent.

Summary Statistics at the bottom:

* Random effects Mean: S M D 0.36, 95 percent C I 0.19 to 0.53, represented by a black diamond on the plot centered at 0.36.

* 95 percent Prediction interval: -0.02 to 0.74, shown as a grey horizontal bar.

* Heterogeneity: tau = 0.15, p = 0.21, I super 2 = 23 percent.

* Total Weight: 100 percent.

The X-axis for the Standardised Mean Difference ranges from -1.5 to 1.5 with a vertical line of no effect at 0. Most studies show a positive S M D, indicating longer completion times for the eating disorder group.

Navigate back to Figure 1.

### Figure 2 Long description

The table contains columns for Study, Vaccine T B and Total, Control T B and Total, R R, 95% C I, Risk Ratio plot, and Weight.

Individual study data includes

* Aronson 1948. R R 0.41, 5% weight.

* Ferguson and Simes 1949. R R 0.20, 6% weight.

* Rosenthal et al. 1960. R R 0.26, 4% weight.

* Hart and Sutherland 1977. R R 0.24, 10% weight.

* Frimodt-Moller et al. 1973. R R 0.80, 9% weight.

* Stein and Aronson 1953. R R 0.46, 10% weight.

* Vandiviere et al. 1973. R R 0.20, 6% weight.

* T B Prev. Trial, Madras 1980. R R 1.01, 10% weight.

* Coetzee and Berjak 1968. R R 0.63, 9% weight.

* Rosenthal et al. 1961. R R 0.25, 8% weight.

* Comstock et al. 1974. R R 0.71, 10% weight.

* Comstock and Webster 1969. R R 1.56, 4% weight.

* Comstock et al. 1976. R R 0.98, 8% weight.

The Risk Ratio plot uses a logarithmic x-axis ranging from 0.1 to 10. A vertical dashed line marks the null effect at 1. Most studies show a risk reduction with squares positioned to the left of the null line.

Summary statistics at the bottom

* Random effects Geometric Mean. 0.49 with 95% C I of 0.34 to 0.70, represented by a black diamond centered at 0.49.

* 95% Prediction interval. 0.13 to 1.78, shown as a horizontal grey bar.

* Heterogeneity. exp tau equals 1.75, p less than 0.01, I-squared equals 92%.

Navigate back to Figure 2.
