# Report tau or exp(tau) rather than tau-squared in random-effects meta-analyses – ADDENDUM

**DOI:** 10.1017/rsm.2026.10095

**Published:** 2026-04-24

**Authors:** Mark D. Chatfield, Louise Marquart-Wilson, Annette Dobson, Daniel Farewell

After acceptance, the authors provided the code and dataset associated with the article to *Research Synthesis Methods* in order to comply with the journal’s control step for reproducibility. The article was passed through that control step.

The code and data should have been published alongside the article, but were inadvertently excluded from the supplementary materials section.

The supplementary materials section of the original article has been updated to include the data and code.

The publisher apologises for the error.
