# Supplementary material for: Report tau or exp(tau) rather than tau-squared in random-effects meta-analyses
Source: Res Synth Methods. 2026 Feb 25;17(4):830–4. doi: 10.1017/rsm.2026.10075 (PMC13311348; doi:10.1017/rsm.2026.10075)
Supplement: Chatfield et al. supplementary material [file S1759287926100751sup001.zip › Appendix.pdf]

Appendix for "Report tau or exp(tau) rather than tau-squared in random-effects meta-analyses", by Chatfield et al.

How τ can help to understand the heterogeneity of ratios

It may be surprising that τ = SD($\log \alpha_i$) is not only helpful to understand the modeled distribution of *log-transformed* true RRs ($\log \alpha_i$). It can also help to understand the modeled distribution of true RRs (α_i). τ can be interpreted as a standard relative deviation,[1] describing variation about the GM. The interpretation is intuitive when τ is small, when a lognormal distribution resembles a normal distribution.

Using the notation of Section 2.2, if GM = 1 and exp(τ) = exp(0.2) = 1.22 then LB1 = 1/1.22 = 0.82 and UB1 = 1.22. Notice how these bounds differ from the GM by approximately $\pm$20% = $\pm\tau$. Similarly, the bounds LB2 = exp(–0.4) = 0.67 and UB2 = exp(0.4) = 1.49 differ from the GM by approximately $\pm$40% = $\pm 2\tau$.

The approximation $\exp(x) \approx 1 + x$ for $|x| \ll 1$ comes from a first-order Taylor series expansion of $\exp(x)$ about $x = 0$. If we consider this approximation acceptable for $|x| \leq 0.4$, then we can appreciate the following about the modeled distribution of true RRs. When $\tau \leq 0.4$, approximately 68% of the distribution lies in the interval [LB1, UB1], where

$$\text{LB1} = \exp(\mu) \times \exp(-\tau) \approx \text{GM} \times (1 - \tau) = \text{GM} - 100\tau\% \times \text{GM}$$

$$\text{UB1} = \exp(\mu) \times \exp(\tau) \approx \text{GM} \times (1 + \tau) = \text{GM} + 100\tau\% \times \text{GM}.$$

When $2\tau \leq 0.4$, approximately 95% of the distribution lies in the interval [LB2, UB2], where

$$\text{LB2} = \exp(\mu) \times \exp(-2\tau) \approx \text{GM} \times (1 - 2\tau) = \text{GM} - 200\tau\% \times \text{GM}$$

$$\text{UB2} = \exp(\mu) \times \exp(2\tau) \approx \text{GM} \times (1 + 2\tau) = \text{GM} + 200\tau\% \times \text{GM}.$$

For larger τ, it is useful to remember that exp(0.7) $\approx$ 2. For example, if τ = 0.35 so that 2τ = 0.7, then the 95% range is approximately [GM/2, GM $\times$ 2] (Table 1).

TABLE 1 Approximate 68% and 95% ranges for selected values of τ.

τ	Approximate 68% range	Approximate 95% range
0	GM $\pm$ 0%	GM $\pm$ 0%
0.1	GM $\pm$ 10%	GM $\pm$ 20%
0.2	GM $\pm$ 20%	GM $\pm$ 40%
0.35	GM $\pm$ 35%	[GM/2, GM $\times$ 2]
0.7	[GM/2, GM $\times$ 2]	[GM/4, GM $\times$ 4]
1.4	[GM/4, GM $\times$ 4]	[GM/16, GM $\times$ 16]

REFERENCES

1. Chatfield MD, Marquart-Wilson L, Dobson AJ, Farewell DM. Mean relative error and standard relative deviation. *Statistica Neerlandica.* 2025;79(1):e70001. doi: 10.1111/stan.70001
